# Ice slurry ingestion reduces human brain temperature measured using non-invasive magnetic resonance spectroscopy

**DOI:** 10.1038/s41598-018-21086-6

**Published:** 2018-02-09

**Authors:** Sumire Onitsuka, Daisuke Nakamura, Takahiro Onishi, Takuma Arimitsu, Hideyuki Takahashi, Hiroshi Hasegawa

**Affiliations:** 1 Graduate School of Integrated Arts and Sciences, Hiroshima University, Higashihiroshima, 739-8521 Japan; 2 Japan Society for the Promotion of Science, Tokyo, 102-0083 Japan; 3 Japan Institute of Sports Sciences, Tokyo, 115-0056 Japan; 4 College of Sport and Health Science, Ritsumeikan University, Kusatsu, 525-8577 Japan

## Abstract

We previously reported that ice slurry ingestion reduced forehead skin temperature, thereby potentially reducing brain temperature (T_brain_). Therefore, in the current study, we investigated the effect of ice slurry ingestion on T_brain_ using proton magnetic resonance spectroscopy, which is a robust, non-invasive method. Eight male participants ingested 7.5 g/kg of either a thermoneutral drink (37 °C; CON) or ice slurry (−1 °C; ICE) for about 5 min following a 15-min baseline period. Then, participants remained at rest for 30 min. As physiological indices, T_brain_, rectal temperature (T_re_), mean skin temperature, nude body mass, and urine specific gravity were measured. Subjective thermal sensation (TS) and thermal comfort (TC) were measured before and after the experiment. T_brain_ and T_re_ significantly reduced after ingestion of ICE compared with after ingestion of CON, and there was a significant correlation between T_brain_ and T_re_. The other physiological indices were not significantly different between beverage conditions. TS and TC were significantly lower with ICE than with CON (*p* < 0.05). These results indicate that ice slurry ingestion can cool the brain, as well as the body’s core.

## Introduction

Ice slurry ingestion, which comprises an icy mixture that is consumed like a beverage^1^, has been recently reported to improve endurance exercise capacity in the heat. Siegel *et al*.^1^ were the first to investigate the effect of ice slurry ingestion on endurance exercise capacity in the heat, and reported that when participants ingested ice slurry (−1 °C) or cold water (4 °C) before exercise, their rectal temperature (T_re_) was significantly reduced, and time to exhaustion improved by 19%. Thereafter, various studies have investigated the effect of ice slurry ingestion in different types of exercise^2,3^, with varied timing of ingestion (e.g., during or after exercise^4–6^), as well as on the central nervous system (e.g., voluntary contraction^7,8^). These studies suggest that ice slurry ingestion has an ergogenic effect. Two mechanisms are proposed as contributing to this effect: (1) increased heat storage capacity through a reduction in core temperature and (2) a sensory effect, such as a reduction in ratings of perceived exertion or an improvement in thermal comfort (i.e., impairment of central fatigue).

Another possible factor for the ergogenic effect of ice slurry ingestion in preventing central fatigue is a reduction in brain temperature (T_brain_). Vanden Hoek *et al*.^9^ compared the core temperatures in swine after central catheter infusions of 50 mL/kg of saline ice slurry and 50 mL/kg of chilled saline, and reported that ice slurry significantly reduced the animals’ T_brain_. In humans, Siegel *et al*.^10^ suggested that oral ingestion of ice slurries possibly resulted in conductive cooling of the facial skin and brain. To verify this hypothesis, we previously investigated the effects of ice slurry ingestion on forehead skin temperature in the heat, and found a significant (vs. 37 °C, *p* < 0.01; vs. 4 °C, *p* < 0.001) reduction in temperature, suggesting a potential reduction in T_brain_ through conductive cooling of the facial skin and brain with ice slurry ingestion^11^. However, our study used an indirect index (i.e., forehead skin temperature). In addition, to the best of our knowledge, no studies have directly observed an alteration in T_brain_ after ice slurry ingestion.

Presently, magnetic resonance spectroscopy (MRS) is utilized as a non-invasive method to measure T_brain_^12–14^. Experimental studies in phantoms used water-N-acetyl aspartate (NAA) solutions as models and revealed a reduction in the difference of NMR frequencies between NAA and water^15,16^. Moreover, experimental models^15–20^ showed a close correlation between temperature that was measured using MRS and those measured using implanted probes. However, this method has only been applied in clinical settings and not in the field of exercise physiology.

Therefore, the aim of this study was to investigate the effect of ice slurry ingestion on T_brain_ using MRS. Given the nature of MRS, we conducted the experiment with participants at rest in a temperate condition.

## Methods

### Participants

Of the 10 healthy men recruited for this study, two were excluded because spectra measurement was difficult (i.e., the participants could not remain still). Therefore, eight healthy men (mean age, 26.9 ± 4.6 y; mean height, 1.71 ± 0.05 m; mean body mass, 67.19 ± 7.80 kg; mean body mass index, 22.9 ± 2.5 kg/m^2^) participa**t**ed in this study. Our study was approved by the Ethics in Human Research Committee of the Japan Institute of Sports Sciences (no. 024), and was performed in accordance with the Declaration of Helsinki. All participants signed an informed consent form prior to participation.

### Experimental design

Our experiment included two separate conditions: ingesting a thermoneutral sports beverage (37 °C; CON) and ingesting ice slurry (−1 °C; ICE). Throughout the study period, participants were asked to maintain their normal lifestyle activities, including their physical activity and nutritional habits. Participants were asked to ingest 500 mL of water 2 h before the experimental trials. Two trials were conducted on 2 consecutive days in a counterbalanced order and at the same time of day to eliminate any potential effects from circadian variations.

Upon arrival in our laboratory, participants’ urine samples were collected, and nude body mass was obtained. Then, a rectal temperature probe was self-inserted, and skin thermistors were attached. Subsequently, subjects put on t-shirts and shorts. Participants entered a room that was maintained at 23 °C with 45% relative humidity (mean room temperature, 23.0 ± 0.2 °C; mean relative humidity, 43.8 ± 1.5%), and were asked to lie supine on the magnetic resonance imaging (MRI) table. The head/neck coil was then attached. After a 15-min rest (baseline period), the participants ingested 7.5 g/kg of either CON or ICE ad libitum over about 5 min (CON, 3.9 ± 1.1 min; ICE, 4.7 ± 1.7 min). After ingestion, participants remained at rest for an additional 30 min.

### Beverage components and protocol

Both beverages were conventional sports drinks (Pocari Sweat; Otsuka Pharmaceutical, Tokyo, Japan) containing 6.2 g of carbohydrates and 49 mg of sodium and 20 mg of potassium per 100 mL as electrolytes. CON was heated in a thermostatic chamber (TR-2A; AS ONE, Osaka, Japan) and ICE was made using a slurry machine (Big Biz1; FMI, Osaka, Japan).

Given the difficulty of consuming beverages while lying supine, participants used a bottle (Floe Bottle; Teknicool Ltd., Auckland, New Zealand) during the ingestion period. The beverage volumes were calculated and prepared by the research staff, who then filled two bottles, each with half of the beverage volume, with the respective beverage (CON or ICE). Before the baseline period, the participants were instructed to pick up one of the two bottles and ingest its contents. Both bottles were then positioned near the participant’s hand immediately before the ingestion period. The participants were instructed to ingest the beverage from either bottle while occasionally mixing the contents of the bottle. The bottles were collected after the contents were consumed. Despite these instructions, some ice remained in the bottle because of its structure; thus, the actual total volume of ingested ICE was lower than 7.5 g/kg. Individual variations were noted for the remaining ice volume, and the mean value for these four participants was 37.50 ± 48.32 g.

### MRI and MRS

**Figure 1:**
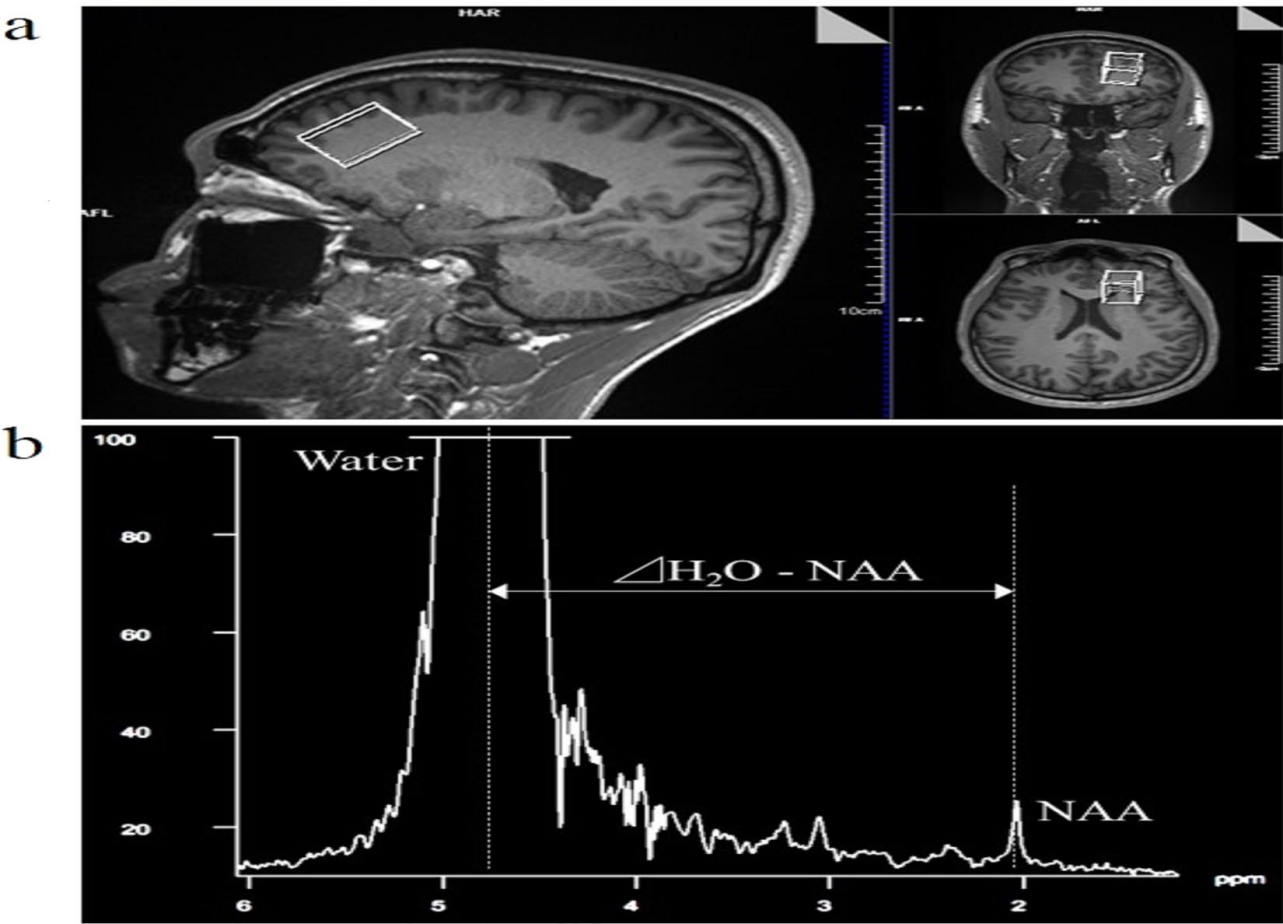
Magnetic resonance spectroscopy. (**a**) Magnetic resonance spectroscopy images of the frontal cortex of interest identified on three-dimensional T1 images. (**b**) An example of the estimation of brain temperature using chemical shift between water and water-N-acetyl aspartate solution.

MRI and MRS were performed using a 3-Tesla MRI scanner (Magnetom Skyra; Siemens Healthcare, Erlangen, Germany) and 64-channel head/neck coil. Three-dimensional (3D) T1-weighted images (magnetisation prepared rapid acquisition with gradient echo) were acquired using the following parameters: repetition time (TR), 1,900 ms; echo time (TE), 2.29 ms; inversion time, 850 ms; flip angle (FA), 8°; matrix, 356 × 256; field of view, 240 mm; and slice thickness, 1 mm^1^. Hydrogen MRS was performed with point-resolved spectroscopy localisation. Acquisition parameters were as follows: TR, 10,000 ms; TE, 135 ms; FA, 90°; data size, 2048; spectral width, 1,200 Hz; and excitation, 1. The voxel of interest (VOI) was set in the frontal cortex, which has been shown to correlate with cognitive function^21^, using the 3D-T1 image (Fig. 1a). The VOI size was 20 × 30 × 20 mm. The magnetic resonance spectrum showed choline-containing compounds at 3.2 ppm, creatine phosphate at 3.0 ppm, and NAA at 2.0 ppm. T_brain_ was calculated from the chemical shift between NAA and water using the equation (1) proposed by Cady *et al*.^12^:

1 $${{\rm{T}}}_{{\rm{brain}}}={\rm{286}}\mathrm{.9}\,-[{\mathrm{94}\times ({\rm{\Delta }}{\rm{H}}}_{2}{\rm{O}}-\mathrm{NAA})],$$

where Δ was the difference between NAA and the water chemical shift (Fig. 1b). We used NAA peak because it is easy to observe and has been used in many earlier studies^12–18^.

### Measurements

As physiological indices, T_brain_, T_re_, mean skin temperature (T_sk_), nude body mass, and urine specific gravity were measured. T_brain_ was measured every 30 s as described above and was averaged every 1 min. The change in T_brain_ (ΔT_brain_) was calculated using the following equation (2):

2 $${\rm{\Delta }}{{\rm{T}}}_{{\rm{brain}}}=\mathrm{mean}\,\,{{\rm{T}}}_{{\rm{brain}}}\,{\rm{after}}\,\,{\rm{beverage}}\,\mathrm{ingestion}\,-{\rm{mean}}\,{{\rm{T}}}_{{\rm{brain}}}\,{\rm{at}}\,\mathrm{Pre},$$

where Pre was defined as the mean T_brain_ of the 15-min baseline period (before beverage ingestion) and after beverage ingestion comprised the 30-min rest period, including the Post measurement. The Post measurement was defined as the end of beverage ingestion (10 min after Pre).

T_re_ was measured with a thermistor (LT-ST08-21; Nikkiso-Therm Co., Ltd., Tokyo, Japan) that was inserted 12 cm from the anal sphincter with a disposable rubber sheath (11Y24; Nikkiso-Therm Co., Ltd.). The change in T_re_ (ΔT_re_) was calculated using the following equation (3):

3 $${\rm{\Delta }}{{\rm{T}}}_{{\rm{r}}{\rm{e}}}={\rm{l}}{\rm{o}}{\rm{w}}{\rm{e}}{\rm{s}}{\rm{t}}\,{\rm{r}}{\rm{e}}{\rm{c}}{\rm{o}}{\rm{r}}{\rm{d}}{\rm{e}}{\rm{d}}\,\,{{\rm{T}}}_{{\rm{r}}{\rm{e}}}\,-{{\rm{T}}}_{{\rm{r}}{\rm{e}}}\,{\rm{a}}{\rm{t}}\,{\rm{P}}{\rm{r}}{\rm{e}},$$

where the time when T_re_ was maximally reduced was defined as that in ICE (23.3 ± 6.9 min), and Pre was defined as the recorded value immediately prior to beverage ingestion (the end of the 15-min baseline period).

Skin temperature was measured at three sites (chest, upper arm, and thigh) using thermistors (LT-ST08-12; Nikkiso-Therm Co., Ltd.) that were secured with micropore tape. All thermistors were connected to a data collection device (LT-8A; Gram Corporation, Saitama, Japan), and temperatures were recorded every 30 s. T_sk_ was calculated using the equation (4) by Roberts^22^:

4 $${{\rm{T}}}_{{\rm{sk}}}=({\rm{0.43}}\,\times \,{\rm{chest}}\,{\rm{temperature}})+({\rm{0.25}}\,\times \,{\rm{arm}}\,{\rm{temperature}})+({\rm{0.32}}\,\times \,{\rm{thigh}}\,{\rm{temperature}}).$$

Nude body mass was measured using a weighing machine (HW-100KGV; A and D, Tokyo, Japan) to the nearest 10 g, and urine specific gravity was determined using a digital urine specific gravity scale (PAL-09S; Atago, Tokyo, Japan) before and after the experiment. Subjective thermal sensation (TS) and thermal comfort (TC) were measured before and after the experiment using the scale derived by Gagge^23^ (0 = very cold to 8 = very hot) and the modified Bedford^24^ scale (1 = very uncomfortable to 7 = very comfortable), respectively. TS and TC of the entire body and head were measured.

### Statistical analysis

**Figure 2:**
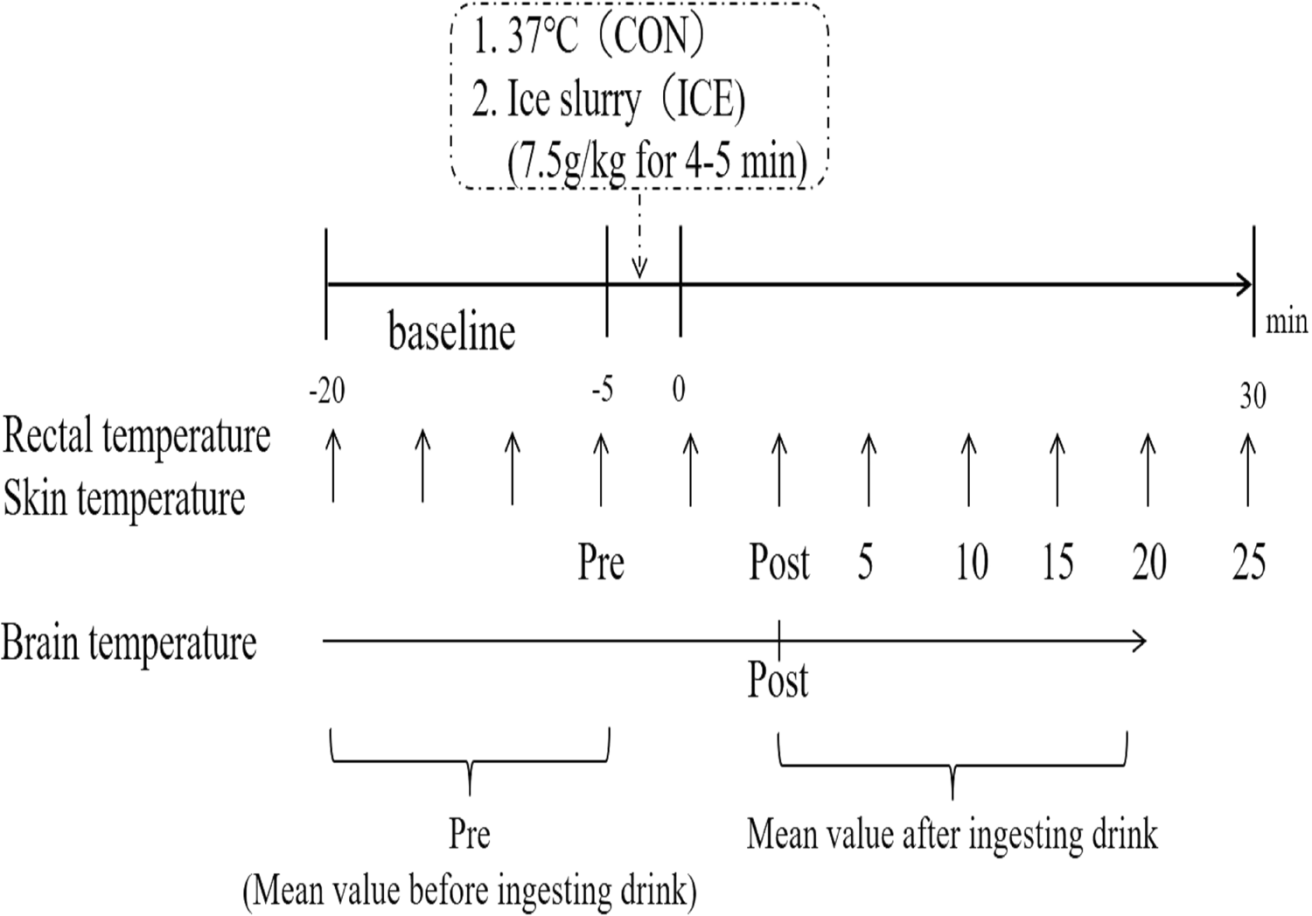
Experimental protocol. A recorded value before beverage ingestion was defined as Pre, and the value at the end of beverage ingestion was defined as Post. For brain temperature, the mean temperature of the 15-min baseline period before beverage ingestion was defined as Pre. All physiological measurements are presented in 5-min increments from Post to 30 min (+30).

Results are presented as mean ± standard deviation. Statistical comparisons of the results for T_brain_, T_re_, T_sk_, TS, TC, body mass, and urine specific gravity were performed using a two-factor (condition × time) analysis of variance with repeated measures. When a significant main effect was identified, the differences were compared using *t*-tests (T_brain_, T_re_, T_sk_, TS, and TC between conditions, and body mass and urine specific gravity before and after the experiment). Pearson’s correlation coefficients were calculated to assess possible correlations between ΔT_brain_ and ΔT_re_. All statistical analyses were performed using SPSS Statistics 17.0 software package (SPSS, Inc., Chicago, IL, USA). Statistical significance was accepted at *p* < 0.05. All physiological measurements after beverage ingestion are presented in 5-min increments from Post to 30 min (+30) (Fig. 2).

### Data Availability

The datasets generated during and/or analysed in the present study are available from the corresponding author on reasonable request.

## Results

### Physiological measurements

**Figure 3:**
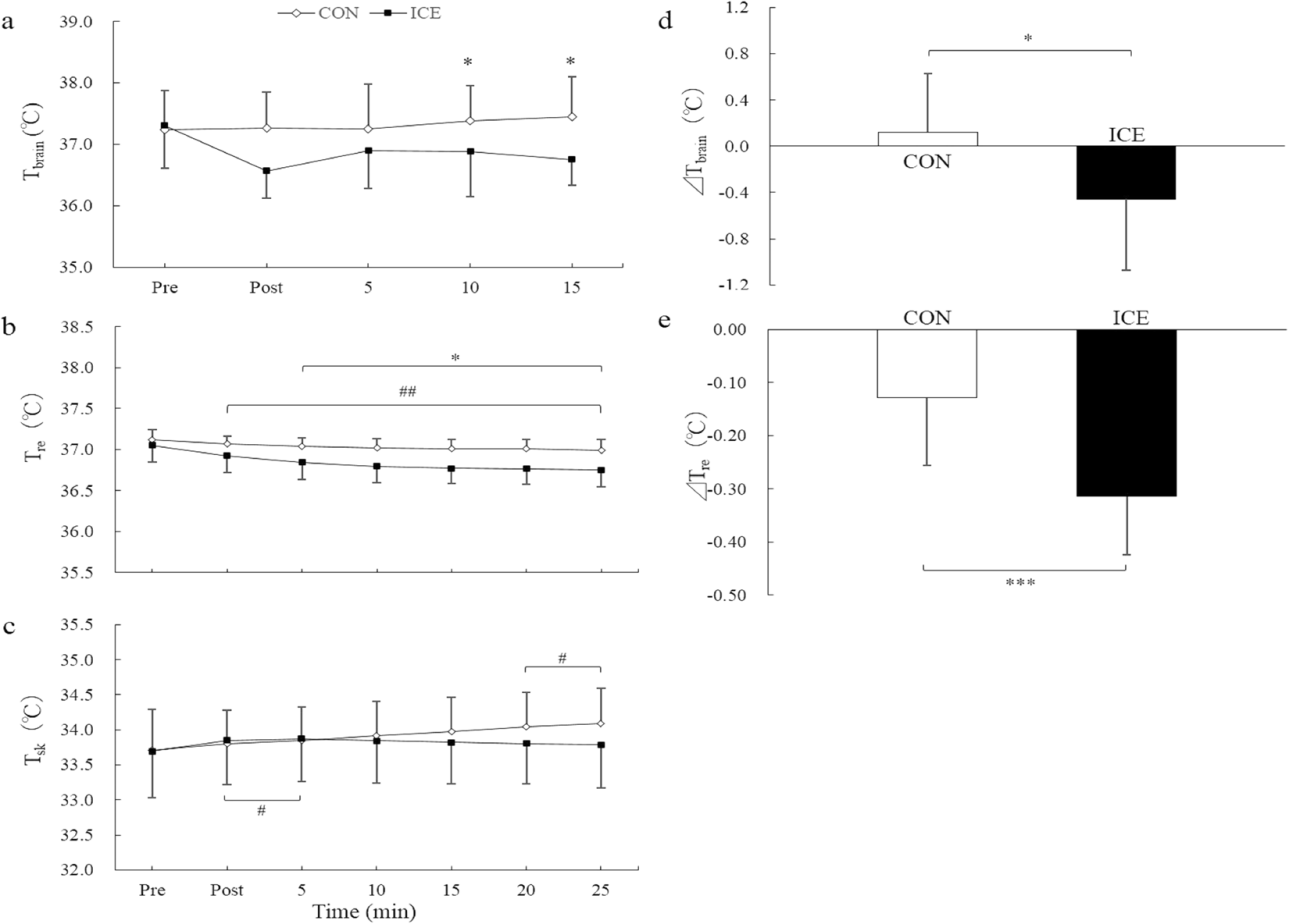
Changes in temperature over time. Changes in (**a**) brain temperature, (**b**) rectal temperature, and (**c**) mean skin temperature during the experiment. Mean changes in (**d**) brain temperature and (**e**) rectal temperature from before and after the experiment. The white rhombus shows CON and the black square shows ICE. Values are expressed as mean ± standard deviation. Significant different between conditions: *p < 0.05; ***p < 0.001. Significantly different than Pre: ^#^*p* < 0.05; ^##^*p* < 0.01.

Changes in temperature over time are presented in Fig. 3. Specifically, changes in T_brain_ during the experiment and ΔT_brain_ are shown in Fig. 3a and d, respectively. ICE had a significantly greater T_brain_ reduction than CON at Post, +10, and +15 (*p* < 0.05 for all). Moreover, ΔT_brain_ was significantly greater in the ICE condition than in the CON condition (*p* < 0.05).

The changes in T_re_ during the experiment and ΔT_re_ are shown in Fig. 3b and e, respectively. T_re_ exhibited a significantly greater reduction with ICE than with CON from +5 to +25 (*p* < 0.05 for all). Additionally, compared to Pre, T_re_ was significantly lower from Post to +25 (*p* < 0.01 for all). Between conditions, ΔT_re_ was significantly greater with ICE than with CON (*p* < 0.001). Further, ΔT_brain_ was significantly correlated with ΔT_re_ (r = 0.60, *p* < 0.05).

Changes in T_sk_ are shown in Fig. 3c. T_sk_ significantly increased at +20 and +25 in the CON condition and at Post and +5 in the ICE condition, but no differences between the conditions were observed.

The hydration state before and after the experiment is summarised in Table 1. In the CON condition, body mass significantly increased after the experiment (*p* < 0.001); however, no significant differences were detected between CON and ICE. Urine specific gravity significantly reduced after the experiment in both conditions (*p* < 0.05), but no significant differences were found between CON and ICE.

**Table 1:** Perception of thermal state and hydration state before and after the experiment.

	CON		ICE	
	Before	After	Before	After
Thermal sensation (entire body)	3.9 ± 0.6	4.5 ± 0.9	3.6 ± 0.5	2.1 ± 0.9***
Thermal comfort (entire body)	4.9 ± 0.8	4.6 ± 1.3	4.5 ± 0.9	2.7 ± 0.5^*,##^
Body mass (kg)	67.00 ± 7.79	67.42 ± 7.84^###^	66.98 ± 7.58	67.34 ± 7.99
Urine specific gravity	1.017 ± 0.009	1.010 ± 0.010^#^	1.013 ± 0.008	1.005 ± 0.002^#^

Values are expressed as mean ± standard deviation. CON, thermoneutral beverage; ICE, ice slurry beverage. Significantly different than CON: *p < 0.05; ***p < 0.001. Significantly different than Before: ^#^*p* < 0.05; ^##^*p* < 0.01; ^###^*p* < 0.001.

### Subjective measurements

Changes in TS and TC are summarised in Table 1. TS of the entire body yielded a significantly greater reduction with ICE than with CON after the experiment (*p* < 0.001). Moreover, ICE yielded a significantly greater reduction in TC of the entire body after the experiment compared to before the experiment (*p* < 0.01). Further, TC of the entire body was significantly reduced in the ICE condition compared with in the CON condition (*p* < 0.05).

## Discussion

The speculation that ice slurry ingestion can impair central fatigue during exercise in the heat by reducing T_brain_ has attracted attention. Some studies have suggested that ice slurry ingestion may reduce T_brain_^25–27^. Because directly measuring T_brain_ in humans is difficult, only an indirect index can be used, particularly during exercise. Therefore, we measured brain temperature at rest using the MRS method to improve the validity of data obtained from the indirect index. Our results showed that ice slurry ingestion significantly reduced T_brain_, as well as T_re_, TS, and TC. These results are important as they provide foundational data for future applied studies.

To the best of our knowledge, the present study is the first to successfully reveal a reduction in T_brain_ in humans through ice slurry ingestion using MRS. Two mechanisms could explain how ice slurry ingestion reduces T_brain_: inflow of cooled carotid blood and conductive cooling of the facial skin and brain^10^. Our previous report of reduced forehead skin temperature with ice slurry ingestion in a warm environment supports the latter mechanism^11^. Given the limitation of MRI, we could not measure forehead skin temperature using a thermistor probe in the present study; however, ice slurry ingestion may possibly reduce facial skin temperature, thereby reducing T_brain_ as previously reported. Interestingly, the reduction in T_brain_ observed in this study (−0.4 °C) was the same as that of head cooling achieved through fanning^28^. Therefore, ice slurry ingestion can cool the brain more easily than fanning, contributing to the development of effective body cooling strategies. Further investigation of this phenomenon is needed.

Fuller *et al*.^29^ reported that despite different pre-exercise temperatures, environmental conditions, and running time, rats reached a point of fatigue at the same T_brain_, suggesting a critical level in T_brain_, as well as core temperature. If the same concept is applied in humans, reaching this critical level can be delayed by pre-cooling the brain with ice slurry ingestion, thereby extending the exercise time. Previous studies reported that a −0.4 °C reduction of core temperature can improve exercise performance^1,27^; hence, a −0.4 °C reduction of T_brain_ may also improve exercise performance. Moreover, the reduction in T_brain_ may explain the ergogenic effect of ice slurry ingestion during exercise. Previous studies reported that ice slurry ingestion during exercise improved performance without changes in core temperature^4,5^. The authors cited an improvement in subjective sensation, such as TC, as the contributing factor. Sensory improvement was possibly influenced by a reduction in T_brain_ as core and skin temperatures were not significantly altered. Although TC became worse with ice slurry ingestion in the present study, we believe that this effect resulted from ingestion in a temperate environment, and heat production was likely not caused by shivering because the mean T_sk_ was unchanged and according to the participants’ perspective.

In previous studies investigating the effects of beverage ingestion on MRI scans^30,31^, the liquid was expelled from a syringe into a capillary tube using a separate syringe pump. However, given that the ice slurry cannot be administered through a tube, the participants in this study picked up the ice slurry bottle by themselves and consumed the beverage ad libitum. Two limitations should be noted for this aspect. First, we cannot exclude the possibility that the movement of the participant’s head disturbed the acquisition of spectra. Indeed, T_brain_ during beverage ingestion was excluded because of spectral issues. However, as the spectra after beverage ingestion was stable, similar to that before beverage ingestion, we believed that it was possible to investigate the effect of ice slurry ingestion on T_brain_. Second, some ice remained in the bottle despite pouring the ice slurry. Hence, we could not control the volume of beverage that the participants ingested. Therefore, ΔT_re_ with ICE was smaller in the present study than that (0.66 °C) in a previous study in a temperate environment^1^. If the participants were able to ingest the required volume, ΔT_re_ with ICE may be greater in the present study. Moreover, the present study found a significant correlation between ΔT_re_ and ΔT_brain_, indicating that the greater the ΔT_re_, the greater the ΔT_brain_; thus, ΔT_brain_ with ICE might also be greater if participants ingest the required volume. Our results are robust because we show that even a lower volume of ice slurry can reduce T_brain_.

An indirect method using tympanic temperature was reported as an alternative to measuring T_brain_ during exercise. However, many studies have demonstrated that this method is challenging, especially during physical exertion in the heat, and can lead to measurement errors caused by dirt, inaccurate placement, and insufficient skill of the measurer^32–36^. Thus, it does not provide an accurate reflection of core temperature^37^. Conversely, measuring T_brain_ before and after exercise using MRS is possible; therefore, investigating the effect of ice slurry ingestion on T_brain_ when the body temperature increases from exercise is also possible.

In the present study, we measured T_brain_ at the frontal cortex, which is related to cognitive function as the index of central fatigue. However, T_brain_ in other brain regions may be different. For example, some studies have reported temperature gradients of up to 1 °C from cooler superficial to warmer deep brain tissues in humans^13^,^38,39^. Therefore, it is worthwhile to measure T_brain_ in other deep regions that are related to thermoregulation, such as the hypothalamus.

Ice slurry ingestion significantly reduced T_brain_, as well as T_re_ and TS in humans. Our findings can contribute to the development of effective body cooling strategies. However, further research is needed to clarify the underlying mechanism of the ergogenic effect of ice slurry ingestion.
